# 2,2-Diphenyl­acetamide

**DOI:** 10.1107/S1600536811026717

**Published:** 2011-07-09

**Authors:** Jerry P. Jasinski, James A. Golen, M. S. Siddegowda, H. S. Yathirajan, M. T. Swamy

**Affiliations:** a Department of Chemistry, Keene State College, 229 Main Street, Keene, NH 03435-2001, USA; b Department of Studies in Chemistry, University of Mysore, Manasagangotri, Mysore 570 006, India; c Department of Chemistry, Sambhram Institute of Technology, Bangalore 560 097, India

## Abstract

In the title compound, C_14_H_13_NO, which has two mol­ecules in the asymmetric unit, the dihedral angles between the mean planes of the benzene rings are 84.6 (7) and 85.0 (6)°. N—H⋯O hydrogen bonds [forming *R*_2_^2^(8) ring motifs] and C—H⋯O hydrogen bonds dominate the crystal packing, forming zigzag chains parallel to the *a* axis. In addition, weak inter­molecular C—H⋯π inter­actions are observed.

## Related literature

For the synthesis and anti­mycobacterial activity of 2,2-diphenyl­acetamide derivatives, see: Guzel *et al.* (2006 ▶). For related structures, see: Akkurt *et al.* (2007 ▶); Dutkiewicz *et al.* (2010 ▶); Gerkin (1998 ▶); Krigbaum *et al.* (1968 ▶); Narasegowda *et al.* (2005 ▶); Yathirajan *et al.* (2005 ▶). For standard bond lengths, see: Allen *et al.* (1987 ▶).
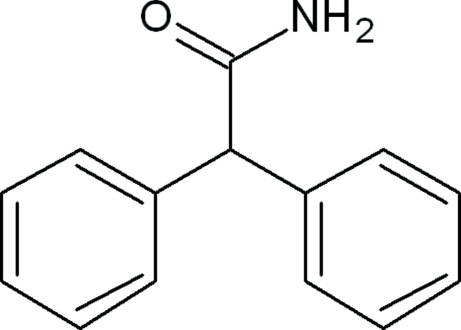


## Experimental

### 

#### Crystal data

C_14_H_13_NO
*M* *_r_* = 211.25
Monoclinic, 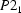

*a* = 5.1687 (3) Å
*b* = 28.5511 (13) Å
*c* = 7.8006 (4) Å
β = 98.152 (5)°
*V* = 1139.52 (10) Å^3^
*Z* = 4
Cu *K*α radiation
μ = 0.61 mm^−1^
*T* = 173 K
0.40 × 0.25 × 0.20 mm

#### Data collection

Oxford Diffraction Xcalibur Eos Gemini diffractometer
Absorption correction: multi-scan (*CrysAlis RED*; Oxford Diffraction, 2010 ▶) *T*_min_ = 0.792, *T*_max_ = 0.887
6490 measured reflections
3978 independent reflections
3869 reflections with *I* > 2σ(*I*)
*R*_int_ = 0.017

#### Refinement

*R*[*F* ^2^ > 2σ(*F* ^2^)] = 0.037
*wR*(*F* ^2^) = 0.107
*S* = 1.07
3978 reflections
303 parameters
9 restraints
H atoms treated by a mixture of independent and constrained refinement
Δρ_max_ = 0.21 e Å^−3^
Δρ_min_ = −0.20 e Å^−3^

### 

Data collection: *CrysAlis PRO* (Oxford Diffraction, 2010 ▶); cell refinement: *CrysAlis PRO*; data reduction: *CrysAlis RED* (Oxford Diffraction, 2010 ▶); program(s) used to solve structure: *SHELXS97* (Sheldrick, 2008 ▶); program(s) used to refine structure: *SHELXL97* (Sheldrick, 2008 ▶); molecular graphics: *SHELXTL* (Sheldrick, 2008 ▶); software used to prepare material for publication: *SHELXTL*.

## Supplementary Material

Crystal structure: contains datablock(s) global, I. DOI: 10.1107/S1600536811026717/jh2307sup1.cif

Structure factors: contains datablock(s) I. DOI: 10.1107/S1600536811026717/jh2307Isup2.hkl

Supplementary material file. DOI: 10.1107/S1600536811026717/jh2307Isup3.cml

Additional supplementary materials:  crystallographic information; 3D view; checkCIF report

## Figures and Tables

**Table 1:** Hydrogen-bond geometry (Å, °) *Cg*1 and *Cg*4 are the centroids of the C3–C8 and C23–C28 rings, respectively.

*D*—H⋯*A*	*D*—H	H⋯*A*	*D*⋯*A*	*D*—H⋯*A*
N1—H1*B*⋯O1^i^	0.89 (1)	2.20 (2)	2.9409 (17)	140 (2)
N1—H1*A*⋯O2^ii^	0.87 (1)	2.09 (1)	2.9575 (19)	177 (2)
N2—H2*B*⋯O1^iii^	0.88 (1)	2.07 (1)	2.9526 (19)	176 (2)
N2—H2*A*⋯O2^iv^	0.89 (1)	2.17 (2)	2.9407 (18)	145 (2)
N1—H1*A*⋯N2^ii^	0.87 (1)	3.06 (2)	3.7246 (18)	134 (2)
N2—H2*B*⋯N1^iii^	0.88 (1)	3.10 (2)	3.7246 (18)	130 (2)
C10—H10*A*⋯O1	0.95	2.50	3.093 (2)	120
C18—H18*A*⋯O2	0.95	2.51	3.099 (2)	120
C2—H2*C*⋯*Cg*1^i^	1.00	2.96	3.9379 (18)	165
C16—H16*A*⋯*Cg*4^iv^	1.00	2.95	3.9263 (18)	166

Symmetry codes: (i) 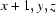; (ii) 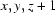; (iii) 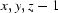; (iv) 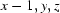.

**Fig. 1.**
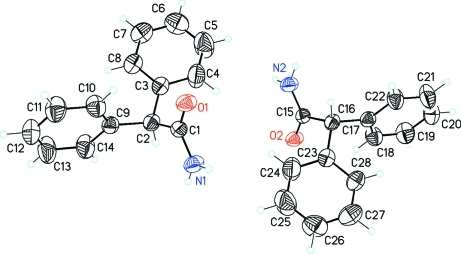
Molecular structure of the title compound showing the atom labeling scheme and 50% probability displacement ellipsoids.

**Fig. 2.**
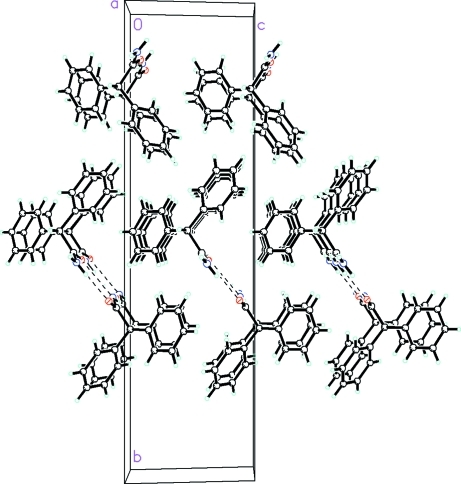
Packing diagram of the title compound viewed down the a axis. Dashed lines represent N—H···O hydrogen bonds.

## supplementary crystallographic information

### Comment

The synthesis and antimycobacterial activity of some new 2,2-diphenylacetamide derivatives is described (Guzel *et al.*, 2006). The title compound is used to synthesize various biologically active and pharmaceutical compounds viz., loperamide, darifenacin, fenpiverine, etc. The crystal structures of N,N-diphenylacetamide (Krigbaum *et al.*, 1968), 4,4'-dimethylbiphenyl-2,2'-dicarboxylic acid (Gerkin, 1998), 4'-methylbiphenyl-2-carboxylic acid (Narasegowda *et al.*, 2005) and 4'-(2-butyl-4-chloro-5-formylimidazol-1-ylmethyl)biphenyl-2-carbonitrile (Yathirajan *et al.*, 2005), 2-hydroxy-N-(3-oxo-1-thia-4-azaspiro[4.5]dec-4-yl)-2,2-diphenylacetamide (Akkurt *et al.*, 2007) and 2-Chloro-N-[4-chloro-2-(2-chlorobenzoyl)phenyl]acetamide (Dutkiewicz *et al.*, 2010) have been reported. In view of the importance of the title compound, (I), C_14_H_13_NO, and in order to determine the conformation of this molecule, a crystal structure determination has been carried out.

In the title compound, (I), with two molecules in the asymmetric unit, the dihedral angle between the mean planes of the benzene rings is 84.6 (7)° or 85.0 (6)°, respectively (Fig. 1).  Extensive  N—H···O (forming an R_2_^2^(8) ring-motif) intermolecular and C—H···O intramolecular hydrogen bonds and weak C—H···Cg π-ring intermolecular interactions (dominate the crystal packing forming a zigzag chain along [010] (Table 1, Fg. 2).

### Experimental

The title compound was obtained as a gift sample from R. L. Fine Chem, Bangalore. X-ray quality crystals were obtained by slow evaporation of 1:1 acetone:methanol solution (m.p.: 430-433 K).

### Refinement

The N–H atoms wwere located by Fourier analysis and refined isotropically with DFIX = 0.87Å.  All of the remaining H atoms were placed in their calculated positions and then refined using the riding model with Atom–H lengths of 0.95Å or 1.00Å (CH).  Isotropic displacement parameters for these atoms were set to 1.19-1.20 (CH), times *U*_eq_ of the parent atom.

### Crystal data

**Table:**

C_14_H_13_NO	*F*(000) = 448
*M**_r_* = 211.25	*D*_x_ = 1.231 Mg m^−^^3^
Monoclinic, *P*2_1_	Cu *K*α radiation, λ = 1.54178 Å
Hall symbol: P 2yb	Cell parameters from 5082 reflections
*a* = 5.1687 (3) Å	θ = 4.6–70.4°
*b* = 28.5511 (13) Å	µ = 0.61 mm^−^^1^
*c* = 7.8006 (4) Å	*T* = 173 K
β = 98.152 (5)°	Block, colorless
*V* = 1139.52 (10) Å^3^	0.40 × 0.25 × 0.20 mm
*Z* = 4	

### Data collection

**Table:**

Oxford Diffraction Xcalibur Eos Gemini diffractometer	3978 independent reflections
Radiation source: Enhance (Cu) X-ray Source	3869 reflections with *I* > 2σ(*I*)
graphite	*R*_int_ = 0.017
Detector resolution: 16.1500 pixels mm^-1^	θ_max_ = 70.5°, θ_min_ = 5.7°
ω scans	*h* = −6→6
Absorption correction: multi-scan (*CrysAlis RED*; Oxford Diffraction, 2010)	*k* = −34→34
*T*_min_ = 0.792, *T*_max_ = 0.887	*l* = −9→8
6490 measured reflections	

### Refinement

**Table:**

Refinement on *F*^2^	Secondary atom site location: difference Fourier map
Least-squares matrix: full	Hydrogen site location: inferred from neighbouring sites
*R*[*F*^2^ > 2σ(*F*^2^)] = 0.037	H atoms treated by a mixture of independent and constrained refinement
*wR*(*F*^2^) = 0.107	*w* = 1/[σ^2^(*F*_o_^2^) + (0.0695*P*)^2^ + 0.1057*P*] where *P* = (*F*_o_^2^ + 2*F*_c_^2^)/3
*S* = 1.07	(Δ/σ)_max_ = 0.027
3978 reflections	Δρ_max_ = 0.21 e Å^−^^3^
303 parameters	Δρ_min_ = −0.20 e Å^−^^3^
9 restraints	Extinction correction: *SHELXL97* (Sheldrick, 2008), Fc^*^=kFc[1+0.001xFc^2^λ^3^/sin(2θ)]^-1/4^
Primary atom site location: structure-invariant direct methods	Extinction coefficient: 0.0118 (13)

### Special details

**Table:**

Geometry. All esds (except the esd in the dihedral angle between two l.s. planes) are estimated using the full covariance matrix. The cell esds are taken into account individually in the estimation of esds in distances, angles and torsion angles; correlations between esds in cell parameters are only used when they are defined by crystal symmetry. An approximate (isotropic) treatment of cell esds is used for estimating esds involving l.s. planes.
Refinement. Refinement of *F*^2^ against ALL reflections. The weighted *R*-factor *wR* and goodness of fit *S* are based on *F*^2^, conventional *R*-factors *R* are based on *F*, with *F* set to zero for negative *F*^2^. The threshold expression of *F*^2^ > σ(*F*^2^) is used only for calculating *R*-factors(gt) *etc*. and is not relevant to the choice of reflections for refinement. *R*-factors based on *F*^2^ are statistically about twice as large as those based on *F*, and *R*- factors based on ALL data will be even larger.

### Fractional atomic coordinates and isotropic or equivalent isotropic displacement parameters (Å^2^)

**Table:**

	*x*	*y*	*z*	*U*_iso_*/*U*_eq_	
N1	0.9797 (3)	0.54261 (6)	0.6365 (2)	0.0459 (3)	
H1B	1.121 (3)	0.5307 (8)	0.600 (3)	0.055*	
H1A	0.984 (5)	0.5667 (6)	0.706 (3)	0.055*	
O2	1.0081 (2)	0.62240 (4)	−0.11977 (17)	0.0465 (3)	
O1	0.5496 (2)	0.53784 (5)	0.64407 (16)	0.0459 (3)	
N2	0.5779 (3)	0.61736 (6)	−0.1120 (2)	0.0469 (4)	
H2B	0.561 (5)	0.5935 (6)	−0.185 (3)	0.056*	
H2A	0.431 (3)	0.6281 (9)	−0.080 (3)	0.056*	
C1	0.7494 (3)	0.52226 (5)	0.5965 (2)	0.0359 (3)	
C2	0.7440 (3)	0.47718 (6)	0.4902 (2)	0.0377 (3)	
H2C	0.9097	0.4758	0.4381	0.045*	
C3	0.5167 (3)	0.47744 (6)	0.3416 (2)	0.0406 (4)	
C4	0.4550 (5)	0.51789 (8)	0.2469 (3)	0.0678 (6)	
H4A	0.5534	0.5456	0.2759	0.081*	
C5	0.2520 (6)	0.51860 (10)	0.1106 (4)	0.0824 (8)	
H5A	0.2130	0.5467	0.0469	0.099*	
C6	0.1062 (5)	0.47893 (10)	0.0666 (3)	0.0683 (6)	
H6A	−0.0353	0.4796	−0.0254	0.082*	
C7	0.1684 (4)	0.43851 (9)	0.1575 (3)	0.0577 (5)	
H7A	0.0707	0.4108	0.1272	0.069*	
C8	0.3724 (4)	0.43764 (7)	0.2933 (2)	0.0461 (4)	
H8A	0.4138	0.4092	0.3543	0.055*	
C9	0.7419 (3)	0.43519 (5)	0.6105 (2)	0.0375 (3)	
C10	0.5760 (4)	0.43225 (7)	0.7334 (3)	0.0518 (4)	
H10A	0.4577	0.4571	0.7451	0.062*	
C11	0.5795 (5)	0.39352 (9)	0.8402 (3)	0.0606 (5)	
H11A	0.4630	0.3921	0.9239	0.073*	
C12	0.7488 (5)	0.35715 (8)	0.8270 (3)	0.0607 (5)	
H12A	0.7521	0.3309	0.9020	0.073*	
C13	0.9135 (5)	0.35925 (9)	0.7036 (4)	0.0738 (7)	
H13A	1.0288	0.3340	0.6910	0.089*	
C14	0.9117 (4)	0.39807 (8)	0.5981 (3)	0.0607 (5)	
H14A	1.0294	0.3994	0.5151	0.073*	
C15	0.8083 (3)	0.63780 (6)	−0.0712 (2)	0.0355 (3)	
C16	0.8143 (3)	0.68289 (5)	0.0360 (2)	0.0367 (3)	
H16A	0.6495	0.6841	0.0892	0.044*	
C17	0.8148 (3)	0.72535 (6)	−0.0840 (2)	0.0373 (3)	
C18	0.9825 (4)	0.72830 (7)	−0.2076 (2)	0.0505 (4)	
H18A	1.1005	0.7034	−0.2201	0.061*	
C19	0.9779 (4)	0.76758 (8)	−0.3129 (3)	0.0594 (5)	
H19A	1.0941	0.7694	−0.3967	0.071*	
C20	0.8089 (5)	0.80352 (7)	−0.2976 (3)	0.0585 (5)	
H20A	0.8061	0.8301	−0.3710	0.070*	
C21	0.6427 (5)	0.80100 (8)	−0.1751 (3)	0.0654 (6)	
H21A	0.5257	0.8260	−0.1627	0.078*	
C22	0.6461 (4)	0.76191 (7)	−0.0699 (3)	0.0533 (5)	
H22A	0.5294	0.7603	0.0137	0.064*	
C23	1.0422 (3)	0.68233 (6)	0.1827 (2)	0.0404 (3)	
C24	1.1076 (5)	0.64165 (8)	0.2764 (3)	0.0702 (6)	
H24A	1.0119	0.6137	0.2462	0.084*	
C25	1.3098 (6)	0.64110 (11)	0.4128 (3)	0.0848 (8)	
H25A	1.3508	0.6129	0.4757	0.102*	
C26	1.4513 (5)	0.68078 (11)	0.4580 (3)	0.0696 (6)	
H26A	1.5922	0.6801	0.5505	0.084*	
C27	1.3884 (4)	0.72136 (9)	0.3690 (3)	0.0608 (5)	
H27A	1.4847	0.7491	0.4008	0.073*	
C28	1.1853 (4)	0.72226 (7)	0.2329 (2)	0.0468 (4)	
H28A	1.1432	0.7508	0.1727	0.056*	

### Atomic displacement parameters (Å^2^)

**Table:**

	*U*^11^	*U*^22^	*U*^33^	*U*^12^	*U*^13^	*U*^23^
N1	0.0328 (6)	0.0411 (8)	0.0651 (9)	−0.0033 (6)	0.0110 (6)	−0.0140 (7)
O2	0.0316 (5)	0.0435 (7)	0.0654 (7)	−0.0009 (5)	0.0106 (5)	−0.0159 (5)
O1	0.0321 (5)	0.0424 (6)	0.0639 (7)	−0.0001 (5)	0.0093 (5)	−0.0150 (6)
N2	0.0325 (6)	0.0435 (8)	0.0652 (9)	−0.0021 (6)	0.0086 (6)	−0.0147 (7)
C1	0.0322 (7)	0.0325 (8)	0.0430 (8)	0.0009 (5)	0.0051 (6)	−0.0007 (6)
C2	0.0361 (7)	0.0352 (8)	0.0436 (8)	−0.0012 (6)	0.0116 (6)	−0.0032 (7)
C3	0.0436 (8)	0.0388 (9)	0.0407 (8)	−0.0019 (6)	0.0107 (6)	−0.0039 (7)
C4	0.0871 (15)	0.0441 (11)	0.0663 (13)	−0.0108 (10)	−0.0092 (11)	0.0077 (9)
C5	0.107 (2)	0.0650 (15)	0.0660 (14)	−0.0016 (14)	−0.0202 (14)	0.0137 (12)
C6	0.0713 (13)	0.0847 (16)	0.0442 (10)	0.0011 (12)	−0.0085 (9)	−0.0065 (10)
C7	0.0624 (11)	0.0665 (13)	0.0438 (9)	−0.0133 (10)	0.0061 (8)	−0.0125 (9)
C8	0.0545 (9)	0.0452 (10)	0.0398 (8)	−0.0061 (8)	0.0109 (7)	−0.0068 (7)
C9	0.0368 (7)	0.0332 (8)	0.0423 (8)	−0.0013 (6)	0.0048 (6)	−0.0052 (6)
C10	0.0547 (10)	0.0487 (11)	0.0557 (10)	0.0113 (8)	0.0204 (8)	0.0036 (8)
C11	0.0734 (13)	0.0576 (11)	0.0565 (11)	0.0015 (10)	0.0284 (10)	0.0067 (10)
C12	0.0769 (13)	0.0434 (11)	0.0636 (12)	0.0004 (10)	0.0164 (10)	0.0106 (9)
C13	0.0837 (15)	0.0440 (11)	0.1012 (18)	0.0193 (11)	0.0389 (14)	0.0163 (11)
C14	0.0657 (12)	0.0439 (10)	0.0795 (14)	0.0133 (9)	0.0346 (10)	0.0054 (10)
C15	0.0327 (7)	0.0324 (7)	0.0418 (7)	−0.0002 (6)	0.0066 (6)	−0.0006 (6)
C16	0.0357 (7)	0.0337 (8)	0.0430 (8)	−0.0020 (6)	0.0129 (6)	−0.0041 (7)
C17	0.0363 (7)	0.0358 (8)	0.0399 (7)	−0.0021 (6)	0.0055 (6)	−0.0057 (6)
C18	0.0531 (10)	0.0481 (10)	0.0534 (10)	0.0080 (8)	0.0186 (8)	0.0042 (8)
C19	0.0686 (12)	0.0591 (12)	0.0550 (11)	0.0016 (10)	0.0245 (9)	0.0069 (10)
C20	0.0752 (13)	0.0429 (11)	0.0583 (11)	−0.0010 (9)	0.0123 (10)	0.0104 (8)
C21	0.0786 (15)	0.0409 (10)	0.0818 (15)	0.0171 (9)	0.0294 (12)	0.0066 (10)
C22	0.0597 (10)	0.0404 (9)	0.0655 (11)	0.0073 (8)	0.0291 (9)	0.0050 (8)
C23	0.0451 (8)	0.0399 (9)	0.0381 (7)	−0.0005 (6)	0.0123 (6)	−0.0036 (7)
C24	0.0878 (16)	0.0478 (12)	0.0684 (13)	−0.0088 (11)	−0.0116 (12)	0.0077 (10)
C25	0.109 (2)	0.0685 (16)	0.0669 (14)	0.0064 (15)	−0.0228 (14)	0.0127 (12)
C26	0.0741 (13)	0.0856 (16)	0.0441 (10)	0.0001 (12)	−0.0091 (9)	−0.0043 (11)
C27	0.0643 (12)	0.0720 (14)	0.0462 (9)	−0.0175 (10)	0.0081 (9)	−0.0141 (10)
C28	0.0553 (10)	0.0452 (10)	0.0411 (8)	−0.0090 (8)	0.0112 (7)	−0.0057 (7)

### Geometric parameters (Å, °)

**Table:**

N1—C1	1.322 (2)	C12—H12A	0.9500
N1—H1B	0.889 (13)	C13—C14	1.380 (3)
N1—H1A	0.873 (13)	C13—H13A	0.9500
O2—C15	1.2306 (19)	C14—H14A	0.9500
O1—C1	1.2292 (19)	C15—C16	1.533 (2)
N2—C15	1.324 (2)	C16—C23	1.521 (2)
N2—H2B	0.883 (13)	C16—C17	1.532 (2)
N2—H2A	0.889 (13)	C16—H16A	1.0000
C1—C2	1.529 (2)	C17—C22	1.375 (2)
C2—C9	1.523 (2)	C17—C18	1.387 (2)
C2—C3	1.529 (2)	C18—C19	1.388 (3)
C2—H2C	1.0000	C18—H18A	0.9500
C3—C8	1.382 (2)	C19—C20	1.364 (3)
C3—C4	1.384 (3)	C19—H19A	0.9500
C4—C5	1.384 (3)	C20—C21	1.374 (4)
C4—H4A	0.9500	C20—H20A	0.9500
C5—C6	1.377 (4)	C21—C22	1.384 (3)
C5—H5A	0.9500	C21—H21A	0.9500
C6—C7	1.369 (4)	C22—H22A	0.9500
C6—H6A	0.9500	C23—C28	1.385 (2)
C7—C8	1.385 (3)	C23—C24	1.389 (3)
C7—H7A	0.9500	C24—C25	1.382 (3)
C8—H8A	0.9500	C24—H24A	0.9500
C9—C10	1.377 (3)	C25—C26	1.367 (4)
C9—C14	1.388 (3)	C25—H25A	0.9500
C10—C11	1.383 (3)	C26—C27	1.366 (4)
C10—H10A	0.9500	C26—H26A	0.9500
C11—C12	1.371 (3)	C27—C28	1.384 (3)
C11—H11A	0.9500	C27—H27A	0.9500
C12—C13	1.373 (4)	C28—H28A	0.9500
			
C1—N1—H1B	120.8 (16)	C13—C14—C9	121.52 (19)
C1—N1—H1A	115.9 (16)	C13—C14—H14A	119.2
H1B—N1—H1A	123 (2)	C9—C14—H14A	119.2
C15—N2—H2B	119.8 (16)	O2—C15—N2	122.28 (16)
C15—N2—H2A	123.8 (16)	O2—C15—C16	120.98 (13)
H2B—N2—H2A	116 (2)	N2—C15—C16	116.72 (13)
O1—C1—N1	122.46 (15)	C23—C16—C17	113.57 (13)
O1—C1—C2	121.11 (14)	C23—C16—C15	110.82 (13)
N1—C1—C2	116.42 (14)	C17—C16—C15	109.44 (12)
C9—C2—C1	109.27 (13)	C23—C16—H16A	107.6
C9—C2—C3	113.52 (13)	C17—C16—H16A	107.6
C1—C2—C3	111.11 (13)	C15—C16—H16A	107.6
C9—C2—H2C	107.6	C22—C17—C18	118.40 (16)
C1—C2—H2C	107.6	C22—C17—C16	119.72 (14)
C3—C2—H2C	107.6	C18—C17—C16	121.88 (14)
C8—C3—C4	117.79 (17)	C17—C18—C19	120.03 (18)
C8—C3—C2	121.97 (15)	C17—C18—H18A	120.0
C4—C3—C2	120.21 (16)	C19—C18—H18A	120.0
C5—C4—C3	121.0 (2)	C20—C19—C18	120.89 (19)
C5—C4—H4A	119.5	C20—C19—H19A	119.6
C3—C4—H4A	119.5	C18—C19—H19A	119.6
C4—C5—C6	120.5 (2)	C19—C20—C21	119.51 (19)
C4—C5—H5A	119.7	C19—C20—H20A	120.2
C6—C5—H5A	119.7	C21—C20—H20A	120.2
C7—C6—C5	119.0 (2)	C20—C21—C22	119.9 (2)
C7—C6—H6A	120.5	C20—C21—H21A	120.1
C5—C6—H6A	120.5	C22—C21—H21A	120.1
C6—C7—C8	120.59 (19)	C17—C22—C21	121.31 (19)
C6—C7—H7A	119.7	C17—C22—H22A	119.3
C8—C7—H7A	119.7	C21—C22—H22A	119.3
C3—C8—C7	121.11 (18)	C28—C23—C24	117.35 (17)
C3—C8—H8A	119.4	C28—C23—C16	121.97 (15)
C7—C8—H8A	119.4	C24—C23—C16	120.64 (16)
C10—C9—C14	117.65 (17)	C25—C24—C23	121.1 (2)
C10—C9—C2	122.44 (15)	C25—C24—H24A	119.5
C14—C9—C2	119.91 (15)	C23—C24—H24A	119.5
C9—C10—C11	120.79 (18)	C26—C25—C24	120.5 (2)
C9—C10—H10A	119.6	C26—C25—H25A	119.7
C11—C10—H10A	119.6	C24—C25—H25A	119.7
C12—C11—C10	120.97 (19)	C25—C26—C27	119.4 (2)
C12—C11—H11A	119.5	C25—C26—H26A	120.3
C10—C11—H11A	119.5	C27—C26—H26A	120.3
C11—C12—C13	119.0 (2)	C26—C27—C28	120.4 (2)
C11—C12—H12A	120.5	C26—C27—H27A	119.8
C13—C12—H12A	120.5	C28—C27—H27A	119.8
C12—C13—C14	120.0 (2)	C27—C28—C23	121.22 (18)
C12—C13—H13A	120.0	C27—C28—H28A	119.4
C14—C13—H13A	120.0	C23—C28—H28A	119.4
			
O1—C1—C2—C9	−79.23 (19)	O2—C15—C16—C23	−46.8 (2)
N1—C1—C2—C9	99.42 (17)	N2—C15—C16—C23	135.00 (15)
O1—C1—C2—C3	46.8 (2)	O2—C15—C16—C17	79.24 (18)
N1—C1—C2—C3	−134.55 (16)	N2—C15—C16—C17	−99.00 (16)
C9—C2—C3—C8	−17.6 (2)	C23—C16—C17—C22	−103.20 (18)
C1—C2—C3—C8	−141.21 (16)	C15—C16—C17—C22	132.39 (17)
C9—C2—C3—C4	164.54 (19)	C23—C16—C17—C18	76.54 (19)
C1—C2—C3—C4	40.9 (2)	C15—C16—C17—C18	−47.87 (19)
C8—C3—C4—C5	1.2 (4)	C22—C17—C18—C19	0.2 (3)
C2—C3—C4—C5	179.2 (2)	C16—C17—C18—C19	−179.50 (18)
C3—C4—C5—C6	0.2 (5)	C17—C18—C19—C20	−0.4 (3)
C4—C5—C6—C7	−1.3 (5)	C18—C19—C20—C21	0.6 (4)
C5—C6—C7—C8	0.9 (4)	C19—C20—C21—C22	−0.7 (4)
C4—C3—C8—C7	−1.6 (3)	C18—C17—C22—C21	−0.3 (3)
C2—C3—C8—C7	−179.55 (17)	C16—C17—C22—C21	179.4 (2)
C6—C7—C8—C3	0.6 (3)	C20—C21—C22—C17	0.6 (4)
C1—C2—C9—C10	47.5 (2)	C17—C16—C23—C28	18.5 (2)
C3—C2—C9—C10	−77.1 (2)	C15—C16—C23—C28	142.15 (16)
C1—C2—C9—C14	−132.47 (18)	C17—C16—C23—C24	−164.12 (19)
C3—C2—C9—C14	102.9 (2)	C15—C16—C23—C24	−40.5 (2)
C14—C9—C10—C11	−0.1 (3)	C28—C23—C24—C25	−0.8 (4)
C2—C9—C10—C11	179.91 (18)	C16—C23—C24—C25	−178.3 (2)
C9—C10—C11—C12	0.3 (3)	C23—C24—C25—C26	−0.4 (5)
C10—C11—C12—C13	−1.0 (4)	C24—C25—C26—C27	1.1 (5)
C11—C12—C13—C14	1.6 (4)	C25—C26—C27—C28	−0.8 (4)
C12—C13—C14—C9	−1.4 (4)	C26—C27—C28—C23	−0.4 (3)
C10—C9—C14—C13	0.7 (3)	C24—C23—C28—C27	1.1 (3)
C2—C9—C14—C13	−179.3 (2)	C16—C23—C28—C27	178.60 (17)

### Hydrogen-bond geometry (Å, °)

**Table:**

Cg1 and Cg4 are the centroids of the C3–C8 and C23–C28 rings, respectively.

**Table:**

*D*—H···*A*	*D*—H	H···*A*	*D*···*A*	*D*—H···*A*
N1—H1B···O1^i^	0.89 (1)	2.20 (2)	2.9409 (17)	140 (2)
N1—H1A···O2^ii^	0.87 (1)	2.09 (1)	2.9575 (19)	177 (2)
N2—H2B···O1^iii^	0.88 (1)	2.07 (1)	2.9526 (19)	176 (2)
N2—H2A···O2^iv^	0.89 (1)	2.17 (2)	2.9407 (18)	145 (2)
N1—H1A···N2^ii^	0.87 (1)	3.06 (2)	3.7246 (18)	134.(2)
N2—H2B···N1^iii^	0.88 (1)	3.10 (2)	3.7246 (18)	130.(2)
C10—H10A···O1	0.95	2.50	3.093 (2)	120.
C18—H18A···O2	0.95	2.51	3.099 (2)	120
C2—H2C···Cg1^i^	1.00	2.96	3.9379 (18)	165
C16—H16A···Cg4^iv^	1.00	2.95	3.9263 (18)	166

Symmetry codes: (i) *x*+1, *y*, *z*; (ii) *x*, *y*, *z*+1; (iii) *x*, *y*, *z*−1; (iv) *x*−1, *y*, *z*.

## References

[bb1] Akkurt, M., Karaca, S., Şahin, E., Güzel, Ö., Salman, A. & İlhan, E. (2007). *Acta Cryst.* E**63**, o3379–o3380.

[bb2] Allen, F. H., Kennard, O., Watson, D. G., Brammer, L., Orpen, A. G. & Taylor, R. (1987). *J. Chem. Soc. Perkin Trans. 2*, pp. S1–19.

[bb3] Dutkiewicz, G., Siddaraju, B. P., Yathirajan, H. S., Narayana, B. & Kubicki, M. (2010). *Acta Cryst.* E**66**, o499. 10.1107/S1600536810003375 PMC2979893 21579901

[bb4] Gerkin, R. E. (1998). *Acta Cryst.* C**54**, 1887–1889. 10.1107/s0108270198009597 9921695

[bb5] Guzel, O., Ilhan, E. & Salman, A. (2006). *Monatsh. Chem.* **137**, 795–801.

[bb6] Krigbaum, W. R., Roe, R.-J. & Woods, J. D. (1968). *Acta Cryst.* B**24**, 1304–1312.

[bb7] Narasegowda, R. S., Yathirajan, H. S. & Bolte, M. (2005). *Acta Cryst.* E**61**, o939–o940.

[bb8] Oxford Diffraction (2010). *CrysAlis PRO* and *CrysAlis RED* Oxford Diffraction Ltd, Yarnton, England.

[bb9] Sheldrick, G. M. (2008). *Acta Cryst.* A**64**, 112–122. 10.1107/S0108767307043930 18156677

[bb10] Yathirajan, H. S., Nagaraj, B., Narasegowda, R. S., Nagaraja, P. & Bolte, M. (2005). *Acta Cryst.* E**61**, o1193–o1195. 10.1107/S0108270105001794 15750249

